# Improved Prodigiosin Production by Relieving CpxR Temperature-Sensitive Inhibition

**DOI:** 10.3389/fbioe.2020.00344

**Published:** 2020-06-03

**Authors:** Yang Sun, Lijun Wang, Xuewei Pan, Tolbert Osire, Haitian Fang, Huiling Zhang, Shang-Tian Yang, Taowei Yang, Zhiming Rao

**Affiliations:** ^1^The Key Laboratory of Industrial Biotechnology, Ministry of Education, School of Biotechnology, Jiangnan University, Wuxi, China; ^2^Ningxia Key Laboratory for Food Microbial-Applications Technology and Safety Control, Yinchuan, China; ^3^School of Agriculture, Ningxia University, Yinchuan, China; ^4^Department of Chemical and Biomolecular Engineering, The Ohio State University, Columbus, OH, United States

**Keywords:** CpxR, *Serratia marcescens*, prodigiosin, temperature-sensitive, metabolic engineering

## Abstract

Prodigiosin (PG) is a typical secondary metabolite mainly produced by *Serratia marcescens*. CpxR protein is an OmpR family transcriptional regulator in Gram-negative bacteria. Firstly, it was found that insertion mutation of *cpxR* in *S. marcescens* JNB 5-1 by a transposon Tn5G increased the production of PG. Results from the electrophoretic mobility shift assay (EMSA) indicated that CpxR could bind to the promoter of the *pig* gene cluster and repress the transcription levels of genes involved in PG biosynthesis in *S. marcescens* JNB 5-1. In the Δ*cpxR* mutant strain, the transcription levels of the *pig* gene cluster and the genes involved in the pathways of PG precursors, such as proline, pyruvate, serine, methionine, and S-adenosyl methionine, were significantly increased, hence promoting the production of PG. Subsequently, a fusion segment composed of the genes *proC, serC*, and *metH*, responsible for proline, serine, and methionine, was inserted into the *cpxR* gene in *S. marcescens* JNB 5-1. On fermentation by the resultant engineered *S. marcescens*, the highest PG titer reached 5.83 g/L and increased by 41.9%, relative to the parental strain. In this study, we revealed the role of CpxR in PG biosynthesis and provided an alternative strategy for the engineering of *S. marcescens* to enhance PG production.

## Introduction

Prodigiosin (PG), a red-colored tripyrrole, has gained increased interest because of its immunosuppressive, anticancer activity. It also has antifungal, antibacterial, antiprotozoal, and antimalarial properties; hence, it has a high potential for pharmaceutical application (Gastmeier, 2014; Gupta et al., 2014). Moreover, it is a good alternative to synthetic colorants and a promising source of food colorants. Prodigiosin is a typical secondary metabolite mainly produced by *Serratia marcescens* (Williamson et al., 2006; Papireddy et al., 2011), which usually appears in the later stages of bacterial growth (Williams, 1973). Incubation temperature plays an important role in PG synthesis in *S. marcescens*. PG could be efficiently produced at 28°C and sharply reduced at 37°C or higher (Williams et al., 1971; Tanaka et al., 2004).

The gene cluster for the biosynthesis of PG has been identified in *Serratia*, and most genes in the cluster have been functionally assigned. The bifurcated pathway culminates into two enzymatically condensed terminal products, 2-methyl-3-n-amyl-pyrrole (MAP) and 4-methoxy-2,2′-bipyrrole-5-carbaldehyde (MBC), and the *pig* clusters *pigD, pigE*, and *pigB* encode proteins that are involved in the production of MAP, while *pigA* and *pigF*–*pigN* encode proteins responsible for the synthesis of MBC (Grimont and Grimont, 1978; Von Graevenitz, 1980; Harris, 2004; Williamson et al., 2006). Proline is converted to 4-hydroxy-2,2′-bipyrrole-5-carbaldehyde (HBC) through a six-step reaction prior to methylation of the HBC hydroxyl group by S-adenosyl methionine-dependent PigF, resulting in the formation of MBC.

Previous studies have largely focused on quorum sensing (Matilla et al., 2015), signal transduction (Fineran et al., 2005), and two-component regulation systems (Horng et al., 2010) to determine the regulatory mechanisms which play an important role in the production of PG. But the thermoregulated mechanism of the production of this secondary metabolite is yet to be elucidated. Moreover, bacteria have evolved a wide variety of signal transduction systems in order to cope with changes in the external environment to ensure their survival and reproduction. This regulatory system has been divided into three categories according to the number of components involved: one-component regulatory system, two-component regulatory system (TCS), and three-component regulatory system (Marijuán et al., 2010). The *cpx* system is a canonical TCS, broadly conserved among Gram-negative bacteria, which consists of the sensor histidine kinase protein CpxA and the regulatory protein CpxR. Studies have suggested the role of CpxA in detecting changes in the external environment, including pH and the overexpression of envelope proteins (such as NlpE or pili subunits), as well as toxic concentrations of metal ions (Nakayama and Watanabe, 1995; Hunke et al., 2012; May et al., 2019). The detection of these stress signals activates CpxA, leading to autophosphorylation at the conserved 151 histidine residue. The histidine residue then carries a phosphate group and transfers it to the aspartic acid residue at position 51 of the response regulatory protein CpxR, hence activating CpxR (Yamamoto and Ishihama, 2006; MacRitchie et al., 2008). The phosphorylated CpxR finally binds to the specific sequence of the target gene promoter and initiates transcription. In this study, we firstly found that there was an increase in the proline, pyruvate, serine, and methionine biosynthetic pathway in the Δ*cpxR* mutant strain, which was beneficial to PG production. So, in this study, we discuss the effects of CpxR on prodigiosin production and further details of its mechanism function.

## Materials and Methods

### Bacterial Strains and Growth Conditions

*S. marcescens* JNB5-1 mutants derived from *S. marcescens* JNB 5-1 were grown in Luria–Bertani (LB) medium or fermentation medium. *Escherichia coli* BL21 (DE3) and *E. coli* DH5α, selected for the expression host, and the *E. coli S17-1* λ*pir* that pUT-Km replicated in were cultured in LB medium. The antibiotics used for selection in *E. coli* or *S. marcescens* JNB 5-1 were ampicillin (Amp), Kan, Chl, apramycin (Apm), clindamycin (Cli), and gentamicin (GM) at concentrations of 100, 50, 25, 50, 100, and 25 μg/ml, respectively. All incubations were performed either at 30 or 37°C. The strains in this work are listed in Supplementary Table S1.

### Tn5G Transposon Mutagenesis

The method used for mutagenesis was the same as that in our previous research (Pan et al., 2019).

### Measuring the Promoter of the *pig* Gene Cluster

Promoters of different lengths were amplified by PCR, and the plasmid pKK 232-8 (or pKKG) was linearized by double-enzyme digestion, followed by recombination of the linear DNA fragment obtained above by T4 ligase (Takara). Then, the recombinant plasmid pKK 232-8 (or pKKG) was transformed into *E. coli DH5*α competent cells, positive clones were selected, and successful construction was verified.

### Measuring the Production of PG

The fermentation broth was dissolved in acidic ethanol (pH 3.0), moderately diluted (50-, 250-, and 1,000-fold), and the diluted sample was sealed and stored for 8 h (fully dissolved), centrifuged at 3,276 × g for 10 min, and the supernatant was taken. OD_535_ was determined. The blank control is acidic ethanol (Kalivoda et al., 2010).

### Assay of PigF

A general procedure for measuring PigF activity was adopted from previous studies (Arnow, 1937; Dhar and Rosazza, 2000). The PigF assay buffer was made of 0.05 M Na_2_HPO_4_-KH_2_PO_4_ (pH 7.0). The assay mixture was composed of the assay buffer containing 1 mM dithiothreitol (DTT), 10 mM MgCl_2_, 2 mM S-adenosyl methionine, 1 mM 4-hydroxy-2,20-bipyrrole-5-carbaldehyde, and 2.5 mg of the enzyme in a final volume of 1 ml. The enzyme reaction mixtures were incubated at 30°C for 25 min before being terminated by the addition of 1 ml of 0.5 M HCl, followed by the addition of 1 ml each of 10% NaNO_2_, 10% NaMoO_4_, and 1 M NaOH. The absorbance at 510 nm of this solution was immediately measured to determine the amount of substrates consumed in the reaction.

### CAT and GFP Fluorescence Experiments

The activity of the chloramphenicol acetyltransferase (CAT) enzyme was measured using Shaw's method, with slight modifications (Shaw, 1975). The reaction system was made of 250 mmol L^−1^ Tris-HCl (pH 7.8), 0.1 mmol L^−1^ acetyl-CoA, 0.4 mg mL^−1^ DTNB, and an appropriate amount of crude enzyme solution. The above reaction mixture was incubated for 2 min in 37°C water, and chloramphenicol was added to a final concentration of 0.1 mmol L^−1^. The light absorption value of A412 was measured immediately after mixing. The reaction solution without chloramphenicol was used as a control.

For the green fluorescent protein (GFP) fluorescence experiments, the BL21 (DE3)-containing plasmid with GFP was cultured in a 24-well plate at 37°C, 180 rpm, for 12 h. The fluorescence intensity (the excitation/emission wavelengths were 480/510) was measured and the BL21 (DE3)-containing plasmid without GFP was used as a control.

### Construction of Mutants

Allelic replacement of the gene open reading frame (ORF) was performed by plasmid pUT-Km, which is a suicide plasmid with a π-protein-dependent origin of replication from the R6K plasmid, so the plasmid can only be replicated in strains expressing the π protein, such as λpir-lysogenic *E. coli S17-1* λ*pir*, SM10(λpir) (de Lorenzo et al., 1990).

For gene knockout, the upstream (containing 9 bp of gene to be knocked out) and downstream (containing 9 bp of gene to be knocked out) homology arms of the gene to be knocked out and the resistance marker gene were amplified by PCR (Supplementary Figure S1). The plasmid pUT-Km is linearized by double-enzyme digestion and recombination of the linear DNA fragment obtained above using the ClonExpress II One Step Cloning Kit (Vazyme, Nanjing, China). Then, the recombinant plasmid pUT-Km was transformed into *E. coli S17-1* λ*pir* competent cells, positive clones were selected, and successful construction was verified.

For gene knock-in, the knockout method was slightly modified, with harvest and amplification of the upstream homology arms, downstream homology arms, and resistance genes, but should amplify a gene to be replaced in the knock-in method and then homologous recombination of the upstream homology arm, replacement gene, resistance marker gene, and the downstream homology arm. The recombinant plasmid pUT-Km was mobilized into *S. marcescens* JNB 5-1 by conjugation. Transconjugants grown on the LB plates contain both Apm (50 μg/ml) and Cli (100 μg/ml) (Maseda et al., 2009). Mutant candidates were screened by colony PCR using primer pairs, which are listed in Supplementary Table S3 (Zhang et al., 2010). The construction of mutants that replaced the *cpxR* loci in *S. marcescens* JNB5-1 was done as described above, and the primers used are listed in Supplementary Table S3. The recombinant strain was obtained by screening on the agar plates containing Apm and Chl, with the results showing that the gene was successfully inserted into the gene of *S. marcescens* JNB 5-1.

### RNA Extraction and Quantitative Real-Time PCR

RNA was prepared from cultures with stationary-phase cultures in LB medium at 30 or 37°C, respectively. Briefly, total RNA was treated with RNase-free pipette tips and RNA was purified using a FastPure Cell/Tissue Total RNA Isolation Kit (Vazyme, Nanjing, China). Of the total RNA, 1 μg was subjected to reverse transcription with 4 μl 5 × HiScript II qRT SuperMix II(Vazyme, Nanjing, China), and the cDNA was subjected to real-time quantitative PCR analysis using the AceQ qPCR SYBR Green Master Mix (Vazyme, Nanjing, China), with the indicated primer pairs listed in Supplementary Table S1. 16S rRNA was used as the internal reference gene and monitored in real time with the StepOnePlus PCR system (Applied Biosystems).

### Electrophoretic Mobility Shift Assay

You's method was slightly modified (You et al., 2018). The *cpxR* gene was amplified with the indicated primers (listed in Supplementary Table S3), cloned into plasmid pET-28a for expression with 6 × His-tag, and then purified. The *pig* gene promoter and target gene ATG front sequences (possible promoter regions) were amplified with the indicated primers (listed in Supplementary Table S3) and ligated to the plasmid pMD19T (Simple) (listed in Supplementary Table S2). Subsequently, the promoter was amplified with the fluorescent-labeled primers M13-47-cy3 and RV-M-Cy3. The purified protein and the Cy3-labeled promoter were incubated in 2 × binding buffer (40 mM Tris-HCl, pH 7.5), 4 mM MgCl_2_, 100 mM NaCl, 10% glycerol, 2 mM DTT, 0.2 mg/ml bovine serum albumin (BSA), 0.02 mg/ml poly(dI-dC), and 1 mM EDTA for 30 min at 25°C. The incubated mixture was electrophoresed on a 5% native polyacrylamide gel electrophoresis (PAGE) for about 1 or 1.5 h. The gel was visualized with ImageQuant LAS 4000 (GE Healthcare Life Sciences, USA).

### Shake Flask Fermentation Assay

The method with our previous research (Pan et al., 2019), with slight modifications. Briefly, JNB5-1 and SMCR were grown at 30°C and 37°C in a rotary shaker with fermentation medium (Sucrose 2%, Beef extract 1.5%, CaCl_2_ 1%, L-proline 0.75%, MgSO_4_·7H_2_O 0.02%, and FeSO_4_·7H_2_O 0.006%) at 180 rpm for 120 h. The production of PG were determined at different fermentation time intervals (0, 12, 24, 36, 48, 60, 72, 84, 96, and 120 h) in triplicate.

### Statistical Analysis

Experiments were done at least twice, with a minimum of three biological replicates. Origin software and TB tools were used for the drawing of the figures, with Student's *t*-tests, Mann–Whitney *U*-test, Fisher's exact test, and one-way ANOVA with Tukey's posttest. Significance was set at a *P-*value of < 0.05

## Results

### PG Production Could Be Enhanced by Disruption of CpxR in *S. marcescens* JNB 5-1

**Figure 1:**
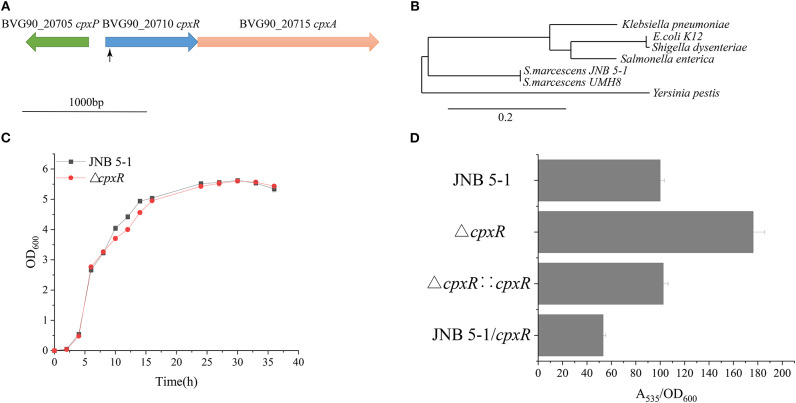
Isolation of increased prodigiosin (PG) production in the Δ*cpxR* mutant. **(A)** Relative positions of *cpxA, cpxR*, and *cpxP* in the CpxARP system in the *Serratia marcescens* UMH8 strain. The locus inserted by the transposon is marked with a *black arrow*. **(B)** Evolutionary relationship with several known proteins among the Enterobacteriaceae. The Phylogram (Dereeper et al., 2008) was drawn from www.Phylogeny.fr in the mode of “one-click” using default setting. The number of substitutions per site is proportional to the length of the branch. **(C)** Growth pattern of JNB 5-1 and the Δ*cpxR* mutant in Luria–Bertani (LB) medium. **(D)** PG yield in JNB 5-1, Δ*cpxR* mutant, complementary strain, and overexpression strain. All the yield displayed by *A*_535_/OD_600_ normalized to JNB 5-1 cultivated at 30°C; the mean ± SD from three independent experiments are shown.

A Tn5G transposon was employed to identify genes, particularly those that influence PG production in *S.marcescens* JNB 5-1. The results from inverse PCR and sequencing of a mutant strain with a significantly increased PG production revealed that transposon insertion into the 38-bp position of a gene in JNB 5-1 could enhance PG production, and the selected gene was later mapped to the BVG90_20710 gene encoding a DNA-binding response regulator on the *S. marcescens* strain UMH8 genome (Figure 1A). Furthermore, bioinformatics analysis predicted that the selected gene from *S. marcescens* JNB 5-1 encodes the two-component regulatory system DNA-binding transcriptional regulator CpxR with a homology of 100% (699/699) to *cpxR* of the *S. marcescens* strain UMH8. At the protein level, the selected gene had a 91.38, 90.95, and 91.38% similarity to the *cpxR* of *E. coli* K12, *Salmonella enterica*, and *Yersinia pestis*, respectively (Supplementary Figure S2). Phylogenetic analysis suggests that the BVG90_20710-encoded protein is structurally intermediate in relatedness between *Y. pestis* and the highly similar *Klebsiella pneumoniae* (Figure 1B).

The Δ*cpxR* mutant displayed a similar growth pattern to that of JNB 5-1 when cultivated in LB medium, indicating that the deletion of *cpxR* had no significant effect on the growth of JNB 5-1 (Figure 1C). Additionally, there was a 76% increase in PG production observed in the Δ*cpxR* mutant compared to *S. marcescens* JNB 5-1, and the complementary strain had restored the level of PG similar to that of JNB 5-1 (Figure 1D). The Δ*cpxR* mutant complementary analysis using the *cpxR* on the JNB 5-1 chromosome further supported the observed phenotypic changes that increased PG production. To confirm that the phenotypic change of JNB 5-1 was due to the loss of *cpxR* or effect on the adjacent genes, the in-frame knockout of *cpxR* was introduced into the JNB 5-1 chromosome by plasmid pUT mini-Km, and the resulting mutant had a similar phenotype to the transposon insertion mutant, resulting in increased PG production. These results indicated that the increase in PG production was due to the mutation of *cpxR* rather than to another mutation or effects on adjacent genes. The Δ*cpxR* mutant phenotypes were also complemented by *cpxR* from *E. coli* K12, *S. enterica*, and *Y. pestis*, with the resulting phenotype showing consistent PG production and growth to that complemented by *cpxR* from JNB 5-1. These results suggest that *cpxR* is highly conserved among the Enterobacteriaceae. Additionally, plasmid-borne overexpression of *cpxR* in the JNB 5-1 strain (with empty plasmid) showed a 46.8% decrease in PG production, indicating the inhibitory effect of *cpxR* (Figure 1D).

### CpxR Binds to the *pig* Gene Cluster Promoter

To further investigate the effect of CpxR on PG production in JNB 5-1, we analyzed the transcription levels of the *pig* gene cluster genes from JNB 5-1 and the Δ*cpxR* mutant by quantitative real-time PCR (qRT-PCR). The results showed that the transcription levels of the *pig* gene cluster genes in the Δ*cpxR* mutant were upregulated (**Figure 4**). This was consistent with the previously discovered phenomenon, which further confirmed that the yield change of PG was influenced by CpxR. We hypothesized that CpxR affected the genes' expressions on the *pig* gene cluster by possibly binding to the *pig* gene cluster promoter.

**Figure 2:**
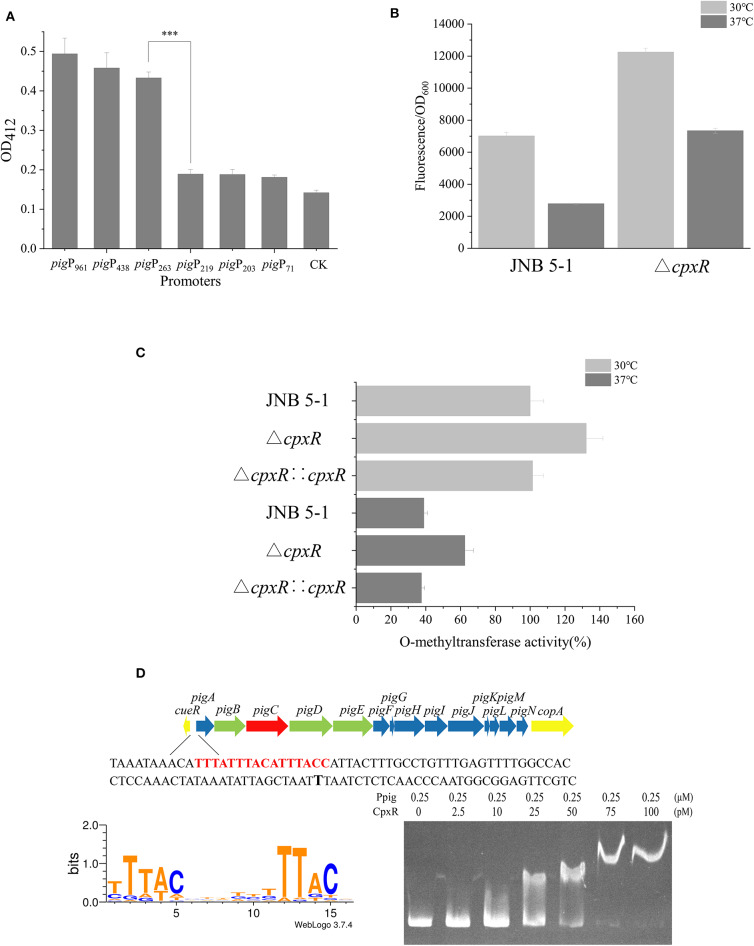
CpxR interaction with the *pig* gene cluster promoter region in JNB 5-1. **(A)** Characterization of promoter activity at different lengths in chloramphenicol acetyltransferase (CAT) activity. **(B)** Green fluorescent protein (GFP) fluorescence intensity of Ppig-*gfp* in JNB 5-1 and the Δ*cpxR* mutant at 30 and 37°C. All the results displayed as fluorescence intensity/OD_600_. **(C)** Whole *O*-methyltransferase activity from the crude cell extract derived from wild-type (with empty plasmid), *cpxR* mutant, and *cpxR* complement strain at 30 and 37°C. All the results displayed as percentage normalized to JNB 5-1 cultivated at 30°C. **(D)** CpxR bound to the *pig* promoter. The *top picture* is the *pig* gene cluster of the 16 genes in *S. marcescens* involved in the prodigiosin biosynthetic pathway. The *yellow block arrow on both ends* indicate the genes that are involved in copper ion transport. The 14 genes in the *middle* are involved in the biosynthesis of prodigiosin, in which the *green block arrow* indicates the genes encoding the protein that synthesizes MAP. *Blue block arrow* indicates the gene encoding the protein that synthesizes MBC, and *red block arrow* shows genes encoding the condensing enzyme in the condensation of MAP and MBC to form prodigiosin. The shown sequence is the front sequence of *pigA* (pig promoter), the *red region* is the CpxR binding region online, and the *black bold T* is the −10 region. The *bottom left picture* is the motif of CpxR harvested online, and *bottom right picture* is the result of the electrophoretic mobility shift assay (EMSA) wherein CpxR bound to the *pig* promoter. The mean ± SD from three independent experiments are shown. Significant differences in activity are calculated by one-way ANOVA. ***P < 0.001.

A promoter plays a critical role in providing transcriptional capacity for nearly every natural and synthetic circuit or pathway (Redden and Alper, 2015). To gain more understanding of the mechanism that CpxR regulates prodigiosin biosynthesis, we constructed a series of recombinant plasmids with different promoter lengths in the putative promoter region of *pigA*. Based on the analysis of the CAT and GFP fluorescence experiments and the Virtual Footprint online software (Munch et al., 2005), the sequence between −263 and the start codon before *pigA* was identified as the promoter of the *pig* gene cluster used in subsequent experiments (Figure 2A and Supplementary Figure S3).

To investigate the effect of CpxR on the transcriptional level of the *pig* gene cluster in JNB 5-1, we constructed a recombinant plasmid carrying the fusion reporter gene (P*pig*-*gfp*, a *pig* gene cluster promoter with *gfp*), as shown in Figure 2B. Evidently, the Δ*cpxR* mutant possessed a higher fluorescence intensity relative to JNB 5-1 at 30 and 37°C, respectively, while the fluorescence intensity of GFP at 30°C was higher than that at 37°C in both the Δ*cpxR* mutant and JNB 5-1, which is consistent with our hypothesis. Specifically, we found that the Δ*cpxR* mutant was 74.6 and 163.5% higher than JNB 5-1 at 30 and 37°C, respectively. However, in the JNB 5-1 and Δ*cpxR* mutants, the fluorescence intensity of GFP at 30°C was 151.9 and 66.9% higher than that at 37°C, respectively. Taken together, these results suggested that temperature had an effect on *cpxR* and modulated the *pig* gene cluster promoter (Figure 2B).

Furthermore, a key *pigF* gene from the *pig* gene cluster encoding *O*-methyltransferase (PigF) was carried out to verify the effect of CpxR on PG biosynthesis in JNB 5-1 at 30 and 37°C. The results showed that *O*-methyltransferase activity was the highest in the Δ*cpxR* mutant compared with JNB 5-1 and the *cpxR* complementary strain at 30 and 37°C (Figure 2C), implying the significant effect of CpxR on *pigF* or the *pig* gene cluster.

EMSA was performed to further gain insight into the regulation mechanism of CpxR on the *pig* cluster promoter. As shown in Figure 2D, the binding affinity increased with an increase in the amount of CpxR protein, suggesting that CpxR bound to the *pig* gene cluster promoter. Interestingly, there was an oblique upward gradient in the binding affinity of CpxR even when the amount of *pig* promoter added is maintained at 0.25 μM. Online analysis on Regulon DB (Santos-Zavaleta et al., 2019) predicted that the CpxR motif is “TTTACNNNNNTTTACN” and binding site was upstream of the *pigA* “TTTATTTACATTTACC” in JNB 5-1 (Figure 2D). The CpxR motif was highly conserved in the *pig* gene cluster promoter when mapping it to the annotated *S. marcescens* (Supplementary Figure S4).

### Inhibition of PG Production by the *cpx* System Corresponds to Temperature in *S. marcescens* JNB 5-1

**Figure 3:**
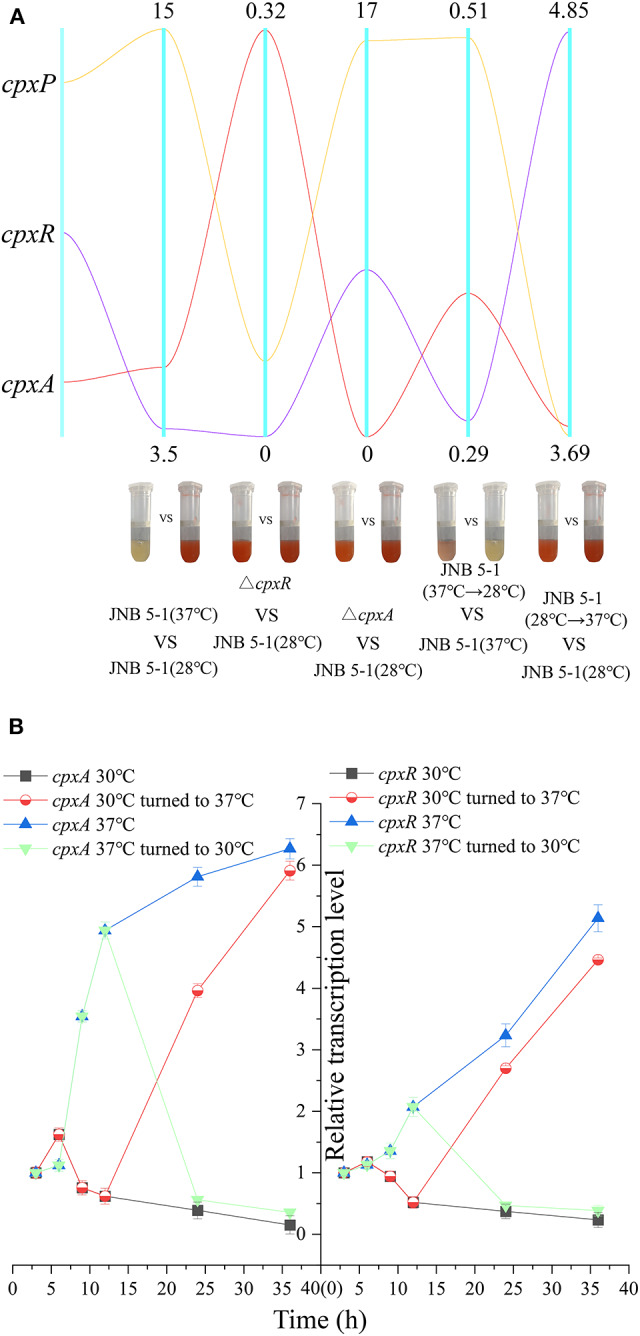
Dynamic analysis of the *cpx* system in response to temperature. **(A)** Dynamic analysis of the *pig* gene cluster and the *cpx* system. The five *vertical line data* represent the comparisons of JNB 5-1 between 30 and 37°C, of the *cpxR* mutant and JNB 5-1 at 30°C, between the *cpxA* mutant and JNB 5-1 at 30°C, between the JNB 5-1 transferred from 37 to 30°C and JNB 5-1 cultivated at 37°C, and between the JNB 5-1 transferred from 30 to 37°C and JNB 5-1 cultivated at 30°C. *Yellow line* represents gene *cpxP, purple line* represents gene *cpxR*, and *red line* represents gene *cpxA*. The *tubes* are the color change of the each strain (meaning change in PG yield). **(B)** Dynamic transcription level analysis of *cpxA* and *cpxR*. qPCR is performed for the characterization of dynamic change of transcription in *cpxA* and *cpxR* in JNB 5-1. The temperatures were altered from 30 to 37°C after 12 h of JNB 5-1 cultivation and *vice versa*. All the values of qRT-PCR were normalized to the first sample (3 h).

Considering the impact of temperature on PG biosynthesis, we investigated the relationship between the *cpx* system and temperature. A qRT-PCR assay was performed on *cpx* for further analysis. As shown in Figure 3A, *cpxR* and *cpxA* were upregulated, with 3.72- and 5.44-fold increases at the transcription level. Subsequently, dynamic transcription-level analysis of the *cpx* system between 30 and 37°C in JNB 5-1 showed a significantly increased transcription level of *cpxA* at 37°C (Figures 3A,B). Initially, there was no change in the transcription level of *cpxR* (0–6 h), but then an increase in the transcription level was observed at the exponential phase (6–12 h), as shown in Figure 3B. This may be attributed to the activation of *cpxR* by CpxA in response to environmental stress. Both *cpxA* and *cpxR* represented a downtrend when the temperature was lowered to 30°C. Subsequently, after 12 h of JNB 5-1 cultivation, the temperatures were altered from 30 to 37°C and *vice versa*, and then dynamic transcription-level tests on *cpxA* and *cpxR* were performed. The results indicated that the transcription level of *cpxA* increased 3.95- and 5.91-fold when cultivated at 37°C after 12 and 24 h, respectively, and decreased by 56.2 and 35.8% when cultivated at 30°C, respectively. A similar trend was observed in *cpxR*, where the transcription level increased by 2.69- and 4.46-fold when turned to 37°C, respectively, and decreased by 46.9 and 34.8% when lowered to 30°C, respectively (Figure 3B). These results suggested that the *cpx* system might affect PG production in the *S. marcescens* JNB 5-1 strain in response to temperature.

Subsequently, we examined the transcript level of the *cpx* system regulatory factor CpxP, which could be activated by CpxR transcription and also acts as an accessory protein to regulate the *cpx* system in the negative feedback loop, to further confirm the relationship between the *cpx* system and temperature. A significant difference in the transcription level of *cpxP* from JNB 5-1 was observed between 30 and 37°C, with the transcription level at 37°C 15-fold higher than that at 30°C. However, the transcription level of *cpxP* in the Δ*cpxR* mutant decreased to 11% when compared to JNB 5-1 cultivated at 30°C, and the transcription level of *cpxP* in the Δ*cpxA* mutant increased by 16.47-fold (Figure 3A). Furthermore, a similar trend to *cpxR* where the transcription level changed in response to temperature was observed, shown in Figure 3A. Taken together, these results suggested that CpxAR-dependent auxiliary protein CpxP expression is temperature-dependent, indicating that there is a substantial impact on CpxAR since the *cpx* system could be activated at 37°C (Figure 3A).

### Δ*cpxR* Mutant Promotes the Supply of PG Synthesis Precursors

**Figure 4:**
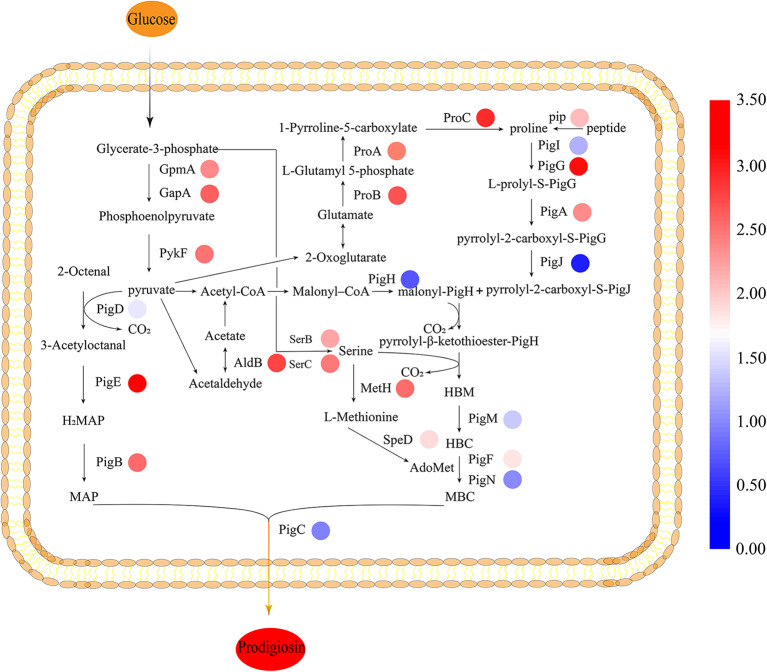
Biosynthesis pathway of prodigiosin in JNB 5-1. All the relative transcription-level values were log_2_-transformed. The biosynthesis of prodigiosin in *Serratia marcescens* is synthesized by a bifurcated pathway of MAP and MBC condensation to form prodigiosin by the condensing enzyme PigC. The *colored rings beside the genes* represent the transcription level, shown as log_2_ (fold change). *MAP*, 2-methyl-3-n-amyl-pyrrole; *MBC*, 4-methoxy-2,2′-bipyrrole-5-carbaldehyde; *AdoMet*, S-adenosyl methionine; *HBC*, 4-hydroxy-2,2′-bipyrrole-5-carbaldehyde; *HBM*, 4-hydroxy-2,2′-bipyrrole-5-methanol. JNB 5-1 and the *cpxR* mutant were cultivated at 30°C.

To gain insight on the effect of CpxR in JNB 5-1 and the changes in the transcription levels of genes on the metabolic pathways involved in PG synthesis in the Δ*cpxR* mutant, we performed qPCR analysis on the genes related to the known precursors for PG synthesis (proline, serine, and l-methionine synthesis) in *S. marcescens*. We found that the transcription levels of the precursors involved in PG synthesis and energy genes were improved. The results showed that the transcript levels of glutamate-5-semialdehyde dehydrogenase (*proA*), glutamate 5-kinase (*proB*), pyrroline-5-carboxylate reductase (*proC*), and proline iminopeptidase (*pip*) were upregulated 4.97-, 5.43-, 6.57-, and 3.23-fold, respectively, in the Δ*cpxR* mutant compared to JNB 5-1, indicating that proline biosynthesis was enhanced in the Δ*cpxR* mutant (Figure 4). Furthermore, the transcription levels of phosphoserine aminotransferase (*serC*) and phosphoserine phosphatase (*serB*), related to serine synthesis, were also upregulated 4.55- and 3.62-fold, respectively, indicating that serine biosynthesis was promoted in the Δ*cpxR* mutant. qRT-PCR analysis also showed that methionine synthase (*metH*) and S-adenosyl methionine decarboxylase (*speD*) were upregulated 4.81- and 2.74-fold, respectively, showing that S-adenosyl methionine, required for the transformation of HBC to MBC, increased in the Δ*cpxR* mutant (Figure 4).

Moreover, the transcription levels of 2,3-bisphosphoglycerate-dependent phosphoglycerate mutase (*gpmA*), glyceraldehyde 3-phosphate dehydrogenase (*gapA*), and pyruvate kinase (*pykF*) related to pyruvate synthesis were upregulated 4.21-, 5.14-, and 4.67-fold, respectively. The transcript level of aldehyde dehydrogenase gene (*aldB*), related to acetaldehyde synthesis, was also upregulated 5.83-fold. Acetaldehyde metabolism is essential for the supply of acetyl-CoA, which could be catalyzed to malonyl-CoA that is beneficial for PG synthesis (Figure 4).

### Reconstruction of *S. marcescens* JNB 5-1 Strain for Efficient Production of Prodigiosin

**Figure 5:**
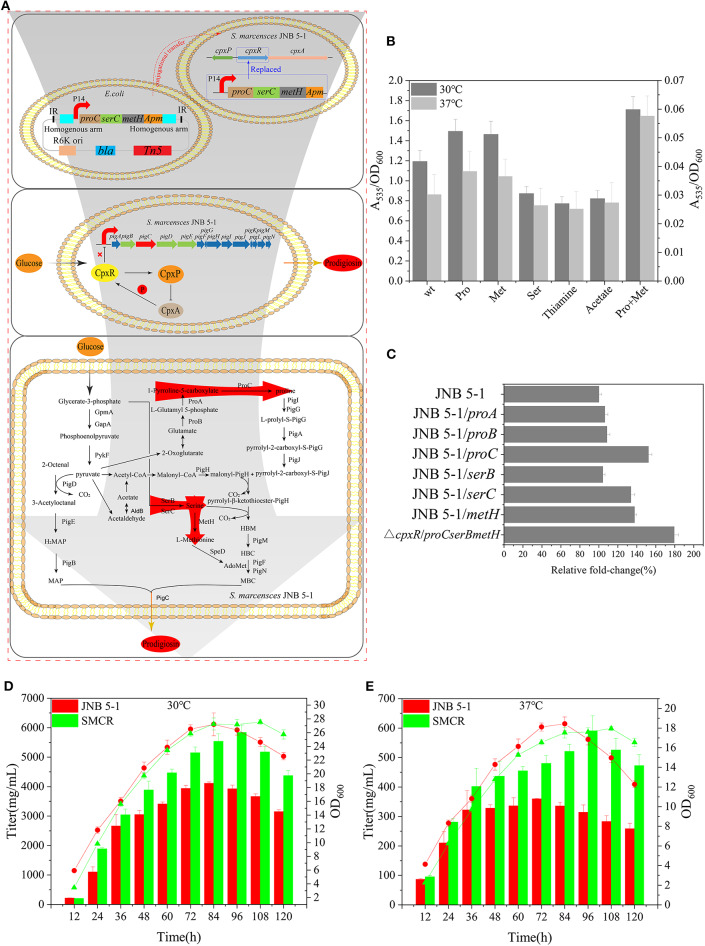
Reconstruction of *Serratia marcescens* JNB 5-1 and fermentation. **(A)** Workflow of the reconstruction of *S. marcescens* JNB 5-1. The *top picture* displays the reconstruction of *S. marcescens* JNB 5-1, replacing *cpxR* with *proC, serC*, and *metH*. The pUT plasmid was used as a knock-in plasmid to send the genes to the JNB 5-1 chromosome by conjugative transfer into JNB 5-1. Subsequently, homologous recombination after entering the cell (*blue box*). The *middle picture* represents the relationship between CpxA, CpxR, and CpxP and the regulation of CpxR on the *pig* gene cluster. The picture displays the biosynthesis of prodigiosin in the hyperproducing *S. marcescens*, in which *cpxR* was replaced by *proC, serC*, and *metH*. *Red arrow region* is the enhanced pathway by overexpression of *proC, serC*, and *metH*. **(B)** Addition of different precursors cultivated in Luria–Bertani (LB) medium at 30 and 37°C. All the yield displayed as *A*_535_/OD_600_. *Left y*-axils and *right y*-axils represent *A*_535_/OD_600_ from strains cultivated at 30 and 37°C, respectively. **(C)** Plasmid-borne overexpression of *proA, proB, proC, serB, serC*, and *metH* in JNB 5-1 in LB cultivated at 30°C. The last result displayed the promotion of mutant replacing the *cpxR* loci by *proC, serC*, and *metH* compared with JNB 5-1. All the yield displayed as *A*_535_/OD_600_ normalized to JNB 5-1 cultivated at 30°C. **(D,E)** Fermentation of mutants and JNB 5-1 cultivated at 30 and 37°C, respectively. *Lines* and *columns* represent OD_600_ and titer, respectively. The means ± SD from three independent experiments are shown.

Proline, serine, and methionine are important factors that promote PG production, considering thiamine and sodium acetate are also beneficial for PG biosynthesis. Therefore, proline, serine, methionine, thiamine, sodium acetate, and a proline–methionine combination were added into the medium for JNB 5-1 growth. The combination of proline–methionine showed the highest PG production relative to the control, and the use of proline or methionine alone also increased PG production (Figure 5B). Subsequently, we performed the plasmid-borne *proA, proB, proC, serB, serC*, and *metH* overexpression on strain JNB 5-1. As anticipated, plasmid-borne *proC, serC*, and *metH* overexpression in strain JNB 5-1 increased by 52.3%, 33.7 and 37.5%, respectively, compared with JNB 5-1 in LB medium harboring empty plasmid (Figure 5C).

Further, in order to overcome the low level of single-copy gene expression by homologous recombination, we integrated the *proC, serC*, and *metH* into the *cpxR* loci (Figure 5A). The titer of PG increased 79% in the recombinant strains with *proC, serC*, and *metH* integrated into the *cpxR* loci (the obtained recombinant strain was renamed SMCH) compared with JNB 5-1 in LB medium (Figure 5C). To fully substantiate the roles of *proC, serC*, and *metH* and *cpxR*, SMCH was subjected to shake flask fermentation for the production of PG in the fermentation medium. Overall, the PG production in SMCH was significantly higher than that of JNB 5-1 at both 30 and 37°C. Specifically, at 30°C, SMCH grew slowly compared to JNB 5-1 at the beginning of growth, and there was no difference between the two strains at the logarithmic phase, as shown in the Figures 5D,E. JNB 5-1 entered the death phase after 84 h, with the stationary period commencing in SMCH and the death phase later beginning at 120 h. Significantly, the highest yield of PG produced in SMCH was 5.83 g/L, which was 41.9% higher than that in JNB 5-1. When cultured at 37°C, there was no significant difference in the growth curve of SMCH and JNB 5-1 from 0 to 84 h, when JNB 5-1 entered the stationary phase. Specific to PG production, SMCH reached the highest yield of 590 mg/L, which was 64.1% higher than JNB 5-1. The results showed that the integration of *proC, serC*, and *metH* into the *cpxR* gene resulted in an increase in the expressions of *proC, serC*, and *metH*, thus improving the production of prodigiosin in *S. marcescens* JNB 5-1.

## Discussion

We herein show a novel insight into the biosynthesis of PG with regulator response to temperature (RRT), in which a two-component regulatory system was thermoregulated and the regulatory protein can bind to the *pig* gene cluster promoter. The Cpx two-component regulatory system that inhibits PG production was screened by transposon insertion, which was thermoregulated and transferred this effect to PG biosynthesis. Subsequently, we reconstructed the key gene of the proline, serine, and methionine metabolic pathway by knock-in and knockout, in which the copy number between *proC* and the Cpx two-component regulatory system changed. This strategy can be used in metabolically engineered strains to improve the copy numbers of *proC, serC*, and *metH*. Lastly, we achieved a hyperproducing *S. marcescens* through the entire genome-modified metabolic strain.

There have been many reports on the effects of the production of PG. Kim et al. improved the prodigiosin production from 0.658 to 1.628 g/L by an antibiotic mutagenesis using chloramphenicol (Kim et al., 2008). Haddix and colleagues harvested the highest production of 1.314 g/L by modifying various fermentation parameters (Haddix and Shanks, 2020); however, the regulatory network may play an important role. The two-component regulation system is an expression system established by bacteria to cope with changes in the external environment (Cheung and Hendrickson, 2010). Previous reports on the Cpx two-component regulatory system have shown that CpxR could regulate the *degP, dsbA*, and *ppiA* genes that encode heat shock protease, disulfide oxidoreductase, and peptide prolyl isomerase, respectively (De Wulf et al., 1999). Our study correlated the Cpx two-component regulatory system to temperature regulation and PG production. The deletion of CpxR resulted in an increase of PG production in JNB 5-1, but did not affect JNB 5-1 growth. However, the overexpression of *cpxR* not only reduced the ability to synthesize PG in JNB 5-1 but also affected the growth of JNB 5-1. Spinola et al. (2010) found that the deletion of *cpxA* led to the accumulation of CpxR in *Haemophilus ducreyi* and weakened its survival (viability) *in vivo*. A similar result was observed, as shown in Figure 3A, where the transcription level of *cpxR* was upregulated in the Δ*cpxA* mutant. Thus, the overexpression of *cpxR* and the deletion of *cpxA* in JNB 5-1 showed that the growth of JNB 5-1 was influenced. García Véscovi Eleonora et al. also found that CpxR expression is temperature-dependent by changes in the *cpxP* transcriptional levels (Bruna et al., 2018). However, we observed that the *cpx* system changed with temperature change (Figure 3A). The transcription levels of the genes in the *pig* gene cluster also changed in *S. marcescens* JNB5-1 and in the Δ*cpxR* and Δ*cpxA* mutants. Furthermore, it was confirmed by EMSA that CpxR bound to the *pig* gene cluster promoter. However, we also noticed that PG production increased in the Δ*cpxR* mutant, but decreased in the Δ*cpxA* mutant, and this could be attributed to the influence of *cpxA* on the growth of *S. marcescens* JNB 5-1 (Delhaye et al., 2016). Moreover, since CpxA is a protein that responds to changes in the surrounding cell membrane environment, such as pH and salt ion concentration, deletion of *cpxA* probably resulted in the inability of cells to adapt to changes in the external environment. We found that *S. marcescens* JNB 5-1 had a highest titer (4.11 g/L) of PG after 84 h, while SMCH produced 5.83 g/L of PG after 96 h. We hypothesized that this was related to the relieved product feedback inhibition due to *cpxR* knockout, which was in agreement with previous reports that CpxR is related to cell membrane biosynthesis (May et al., 2019; Simpson and Trent, 2019).

Proline, synthesized *via* the glutamate pathway by a series of enzymes (ProA, ProB, and ProC), is involved in the first step of MBC synthesis catalyzed by PigG and PigI. It activates l-proline using ATP, resulting in the interaction of l-proline with the thiol group in PigG to form a complex l-prolyl-S-PigG under the action of Pig I, and then PigA oxidizes the l-prolyl-S-PCP to pyrrolyl-2-carboxyl-S-PCP (Williamson et al., 2006). Our research showed that the transcription levels of *proA, proB*, and *proC* involved in proline biosynthesis increased to varying degrees in the Δ*cpxR* mutant, resulting in increased proline synthesis, which was beneficial to PG biosynthesis. This result is in line with a study by Siva et al. that reported enhanced PG production in *Serratia rubidaea* after the addition of proline alone or in combination with methionine (Siva et al., 2012). Moreover, an increased PG production was achieved by plasmid overexpression of *proC* and by adding proline to the fermentation medium in our study. Subsequently, the expression of *proC* was increased by inserting *proC* into the *cpxR* loci, hence a reconstructed *S. marcescens* JNB 5-1 strain with efficient production of prodigiosin.

The transcription levels of the genes involved in pyruvate, serine, and methionine metabolism were also upregulated. Pyruvate is involved in the first step of the MAP branch in the PG biosynthesis bifurcated pathway, which forms 3-acetyloctanal with 2-octenal under the catalysis of PigD and then generates MAP *via* the catalysis of PigE and PigB (Williamson et al., 2006). Wasserman et al. also confirmed the derivation of C2 of MAP, and its attached methyl group is from pyruvate by ^14^C and ^13^C labeling (Wasserman et al., 1973). Initially, we added pyruvate and thiamine to the medium; however, there was no significant increase in PG production. We therefore suspected that pyruvate could be involved in many reactions in the bacteria and was diverted by other metabolic pathways. On the other hand, the maximum rate-limiting factor for PG production could be in the MBC branch. Also, it was predicted that serine involved in the biosynthesis of MBC was condensed with the pyrrolyl-β-ketothioester of PigH, forming 4-hydroxy-2,2′-bipyrrole-5-methanol (HBM) (Williamson et al., 2006), which is an intermediate of HBC required for the synthesis of MBC *via* the action of S-adenosyl methionine-dependent PigF and PigN (Williamson et al., 2005). The replication of the plasmid containing individual genes (*proC, serC*, and *metH*) was carried out by means of circular replication to achieve higher copy numbers. However, the replication generated a large amount of single DNAs, making the plasmid unstable (Aoki et al., 1987; Zhang et al., 2019), thus limiting its industrial application. Therefore, we used integrated expression strategy to insert the gene of the key synthetic enzyme for PG into a certain position on the chromosome of *S. marcescens* JNB5-1, allowing the inserted gene to replicate as the host chromosome replicates. Although the integrated expression level was lower than the plasmid expression level, by increasing the copy number of the integrated *proC*, we were able to achieve high levels of target protein production. In addition, we knocked out a suppressor gene of *S. marcescens* JNB 5-1, resulting in a decreased inhibitory effect on prodigiosin synthesis in *S. marcescens* JNB 5-1 (Zhang et al., 2019). As anticipated, the integration of *proC, serC*, and *metH* into the *cpxR* loci enhanced the transcription and expression of *proC, serC*, and *metH* in *S. marcescens* JNB5-1.

In conclusion, our study demonstrated that the two-component regulatory system *cpx* in *S. marcescens* JNB 5-1 is temperature-regulated and inhibited PG biosynthesis. The proposed RRT system hinted that temperature drives the expression of the *cpx* system, further regulating PG synthesis. The reconstructed high copy numbers of *proC, serC*, and *metH* in *S. marcescens* JNB 5-1 by homologous recombination increased the PG yield.

## Data Availability Statement

All datasets generated for this study are included in the article/Supplementary Material.

## Author Contributions

ZR and TY conceived and designed the study and critically revised the manuscript. TO critically revised the manuscript. YS carried out the experiments, analyzed the data, and drafted the manuscript. LW carried out the experiments. XP, HF, HZ, and S-TY contributed to the revision of the manuscript. All authors read and approved the final manuscript.

## Conflict of Interest

The authors declare that the research was conducted in the absence of any commercial or financial relationships that could be construed as a potential conflict of interest.
