# Supplementary material for: Improved Prodigiosin Production by Relieving CpxR Temperature-Sensitive Inhibition
Source: Front Bioeng Biotechnol. 2020 Jun 3;8:344. doi: 10.3389/fbioe.2020.00344 (PMC7283389; doi:10.3389/fbioe.2020.00344)
Supplement: Supplementary file 1 [file Data_Sheet_1.pdf]

Supplementary files

Supplementary Tables

Supplementary table S1 Strains used in this study

Strains	Description	Source
S. marcescens JNB 5-1	Source of *pig* gene cluster and *cpxARP*, isolated from soil	Lab collection
E. coli BL21 （DE3）	Host for gene expression	Invitrogen
E. coli DH5α	Host for gene expression	Invitrogen
E. coli S17-1 λpir	recA pro hsdR RP4-2-Tc::Mu-Km::Tn7	[1]
△*cpxR*	Deletion *cpxR* in *S. marcescens* JNB 5-1, Apm^R	This work
△*cpxA*	Deletion *cpxA* in *S. marcescens* JNB 5-1, Apm^R	This work
SMCH	*CpxR* loci replaced by *proC*, *serC* and *metH* in JNB 5-1	This work

Supplementary table S2 Plasmids used in this study.

Plasmids	Description	Source
pRK2013 Tn5G	Tn5G carrying plasmid,$Km^R Gm^R$	[2]
pKK 232-8	Amp^R, containing CAT replicate in *E. coli* DH5α	Lab collection
pUT-Km	Amp^R Km^R *ori*R6K *ori*TRP4	[1]
pET-28a (+)	Km^R, replicate in *E. coli* BL21	Novagen
pMD19T (Simple)	A linearized T-vector for cloning promoter fragments, Amp^R	Takara
pKKG	pKK 232-8 derivate, containing gfp replaced CAT replicate in *E. coli* DH5α	This work

pKK961	pKK 232-8 derivate, containing 961bp upstream of *pigA* gene ATG, replicate in *E. coli* DH5α	This work
pKK438	pKK 232-8 derivate, containing 438bp upstream of *pigA* gene ATG, replicate in *E. coli* DH5α	This work
pKK263	pKK 232-8 derivate, containing 263bp upstream of *pigA* gene ATG, replicate in *E. coli* DH5α	This work
pKK219	pKK 232-8 derivate, containing 219bp upstream of *pigA* gene ATG, replicate in *E. coli* DH5α	This work
pKK203	pKK 232-8 derivate, containing 203bp upstream of *pigA* gene ATG, replicate in *E. coli* DH5α	This work
pKK71	pKK 232-8 derivate, containing 71bp upstream of *pigA* gene ATG, replicate in *E. coli* DH5α	This work
pKKG961	pKK961 derivate, containing *gfp* replaced CAT replicate in *E. coli* DH5α	This work
pKKG438	pKK438 derivate, containing *gfp* replaced CAT replicate in *E. coli* DH5α	This work
pKKG263	pKK263 derivate, containing *gfp* replaced CAT replicate in *E. coli* DH5α	This work
pKKG219	pKK219 derivate, containing *gfp* replaced CAT replicate in *E. coli* DH5α	This work
pKKG203	pKK203 derivate, containing *gfp* replaced CAT replicate in *E. coli* DH5α	This work

pKKG71	pKK71 derivate, containing *gfp* replaced CAT replicate in *E. coli* DH5α	This work
pCR	pET-28a derivate, containing *cpxR* replicate in *E. coli* BL21	This work
pCM	pACYC177 derivate, Amp^R Cm^R, replicate in *E. coli*, complementation and overexpression in JNB 5-1	This work
pCMCR	Cm^R, containing *cpxR*, complementation and overexpression in JNB 5-1	This work
pCMPG	Cm^R, *pig* gene promoter P*pig* fused to *gfp*	This work
pCMPA	Cm^R, containing *proA* and overexpression in JNB 5-1	This work
pCMPB	Cm^R, containing *proB* and overexpression in JNB 5-1	This work
pCMPC	Cm^R, containing *proC* and overexpression in JNB 5-1	This work
pCMSB	Cm^R, containing *serB* and overexpression in JNB 5-1	This work
pCMSC	Cm^R, containing *serC* and overexpression in JNB 5-1	This work
pCMMH	Cm^R, containing *metH* and overexpression in JNB 5-1	This work
pKKG	Amp^R, containing *gfp*,replicate in *E. coli* DH5α	This work
pTCR	Amp^R Km^R Apm^R, used for deletion of *cpxR*	This work
pTCA	Amp^R Km^R Apm^R, used for deletion of *cpxA*	This work
pTCCH	Amp^R Km^R Apm^R, containing *proC*, *serC* and *metH* used for replaced of *cpxR*	This work

Amp^R Ampicillin resistant, Km^R kanamycin resistant, Cm^R chloromycin resistant, Apm^R apramycin resistant, Gm^R gentamicin resistant

Supplementary table S3 Primers used in this study.

Primers	Sequence(5'-3')	RS	Note
OTn1	GATCCTGGAAAACGGGAAAG		inverse

			PCR
OTn2	CCATCTCATCAGAGGGTAGT		inverse PCR
cpxR-1 F	cagcaaatgggtcgcggatccATGAACAAGATTCT GTTAGTTGACGAC	*BamH* I	EOC
cpxR-1 R	gcaagcttgtcgacggagctcTCATGTTGCAGATAC CATCAAATAAC	*Sac* I	EOC
cpxR-2 F	cagcaaatgggtcgcggatccATGAATAAAATCCTG TTAGTTGATGATG	*BamH* I	EOC
cpxR-2 R	gcaagcttgtcgacggagctcTCATGAAGCAGAAA CCATCAGATAG	*Sac* I	EOC
cpxR-3 F	cagcaaatgggtcgcggatccGAGGTACGTAAACA ATGAATAAAATCCT	*BamH* I	EOC
cpxR-3 R	gcaagcttgtcgacggagctcTCATGAAGCGGAAA CCATCAG	*Sac* I	EOC
cpxR-4 F	cagcaaatgggtcgcggatccATGCATAAAATCCTA TTAGTTGATGATG	*BamH* I	EOC
cpxR-4 R	gcaagcttgtcgacggagctcTCATGTTTCTGATAC CATCAAGTAGCC	*Sac* I	EOC
pigF F	CAGCAAATGGGTCGCGGATCCATGCCTT TAACCAAGCAAGATGC	*BamH* I	EOC
pigF R	GCAAGCTTGTCGACGGAGCTCTTATTTT TCGCCGACGATCAG	*Sac* I	EOC
proA F	cagcaaatgggtcgcggatccATGCTGGAGCAGAT GGGGA	*BamH* I	EOC
proA R	gcaagcttgtcgacggagctcTTAGCTGCGCACCA GATCG	*Sac* I	EOC
proB F	cagcaaatgggtcgcggatccATGAACGGCAGCCA GACATT	*BamH* I	EOC
proB R	gcaagcttgtcgacggagctcTTAACTGACAATCAT ATCGTCGCG	*Sac* I	EOC
proC F	cagcaaatgggtcgcggatccATGCAACATCGCAA GATTACCTT	*BamH* I	EOC
proC R	gcaagcttgtcgacggagctcTTAAAATAATTTTTC CATCTCTTGGG	*Sac* I	EOC
secB F	cagcaaatgggtcgcggatccATGTCAAACAGTCT GACCTATTGCG	*BamH* I	EOC
secB R	gcaagcttgtcgacggagctcTTATCGCACTTCGTG TTTCAGG	*Sac* I	EOC
secC F	cagcaaatgggtcgcggatccATGACTCAGGTTTAT AATTTTAGCTCTGG	*BamH* I	EOC
secC R	gcaagcttgtcgacggagctcTCAACCGTGACGAC GTTCG	*Sac* I	EOC
metH F	cagcaaatgggtcgcggatccTCAGTCGGCGTCGT	*BamH* I	EOC

	AACCG		
metH R	gcaagcttgtcgacggagctcGTGACGAATCGAGTAGAACAACTGC	*Sac* I	EOC
cpxR U F	acagccggatccccgggtaccTCACTTCTGGGCAGAAGCTTG	*kpn* I	RUHA
cpxR U R	ggttccgcgCTTGTTCATTATGATTTACCTCCAGAC		RUHA
Apm (*cpxR*) F	taatgaacaagCGCGGAACCCCTATTTGTT		Apm
Apm (*cpxR*) R	tcatgttgcTCAGCCAATCGACTGGCG		Apm
cpxR D F	cgattggctgaGCAACATGATCAACAGTTTGACG		RDHA
cpxR D R	gcctaggccgaattcgagctcCGCTGCGCTTTACATTTGG	*Sac* I	RDHA
cpxA U F	acagccggatccccgggtaccCGTCGAACATATGGTGCTGGCAAGCTTG	*kpn* I	RUHA
cpxA U R	gttccgcgGTTGATCATGTTGCAGATACCATCA		RUHA
Apm (*cpxA*) F	aacatgatcaacCGCGGAACCCCTATTTGTT		Apm
Apm (*cpxA*) R	actgttcTCAGCCAATCGACTGGCG		Apm
cpxA D F	gtcgattggctgaGAACAGTAAGAGTTTGCTATCCTGCG		RDHA
cpxA D R	gcctaggccgaattcgagctcTCTATTCAGGCGGTGTTTGGC	*Sac* I	RDHA
proC (*cpxR*) F	tgaacaagATGCAACATCGCAAGATTACCTT		proC
proC (*cpxR*) R	ctgagtcatGAAGTGGTCCTTCTCCTTAACTCG		proC
serC (*cpxR*) F	aggaccacttcATGACTCAGGTTTATAATTTTAGCTCTGG		serC
serC (*cpxR*) R	cgccgactgaGTGAACAAAACTCTCCAATTCTGATAA		serC
metH (*cpxR*) F	ttttgttcacTCAGTCGGCGTCGTAACCG		metH
metH (*cpxR*) R	cgGTGACGAATCGAGTAGAACAACTGC		metH
cueR-F	GGAATAATGGGCAATAACC		RT-qPCR
cueR-R	TTAACCAGCAAGACGATT		RT-qPCR
pigA-F	TTATGCCATGAAATCAAC		RT-qPCR
pigA-R	CATAGACGATGAAGATGT		RT-qPCR
pigB-F	CGGTCTGAGCGGCGTATA		RT-qPCR

pigB-R	CAGCGGAGCAGCAAGAAG	RT-qPCR
pigC-F	GCGATTGGCAGATTTATATGG	RT-qPCR
pigC-R	ATTGTCGGAGCATGTAGG	RT-qPCR
pigD-F	GTCATCAATACCTATCAG	RT-qPCR
pigD-R	TCTGGATATGGAAGTTAT	RT-qPCR
pigE-F	CGAAATGTTCTGCATGAAAT	RT-qPCR
pigE-R	CTGTTGGCGGTAATGAAG	RT-qPCR
pigF-F	TTTCTGTTGAGCGACGAA	RT-qPCR
pigF-R	CCATAGCGGATACAGGAAG	RT-qPCR
pigG-F	CCCGTTGTTTGAACTGAA	RT-qPCR
pigG-R	CGACATGAGACTCCTGAC	RT-qPCR
pigH-F	CGGAACACTTCAATATGA	RT-qPCR
pigH-R	ATAGGAGTTGGAGTAGAA	RT-qPCR
pigI-F	CTGGACGACATGGTGAAA	RT-qPCR
pigI-R	AGGGATGGTGATGAAGGA	RT-qPCR
pigJ-F	TGGCACAAGACTGTTTTC	RT-qPCR
pigJ-R	GTTGATGGTGGAAGCATT	RT-qPCR
pigK-F	CGCCGTTTCACTTTATCG	RT-qPCR
pigK-R	ACTTCCGCCAACTGATTA	RT-qPCR
pigL-F	CGATTGGGACACATACAG	RT-qPCR
pigL-R	CAATTCGTAGGGCAAACC	RT-qPCR
pigM-F	CCTACGCCAATCAATGAA	RT-qPCR
pigM-R	TGCGATACTGAGTGAACT	RT-qPCR
pigN-F	CCCAAAAGCGAGAAAATG	RT-qPCR
pigN-R	AAAGGAATGAAACACTTAACC	RT-qPCR
copA-F	GGATGCGGTGGAACTGTC	RT-qPCR
copA-R	ATGCCCAGCGTATTGTAGAT	RT-qPCR
cpxA-F	CGCCCTGCGGTATTCACA	RT-qPCR
cpxA-R	GCCTCATCGGTGCGGTAGAA	RT-qPCR
cpxR-F	ACCGAGTTCACCCTCCTTTAC	RT-qPCR
cpxR-R	CGCCGCAGGTTGGAGATA	RT-qPCR
cpxP-F	GCTACATTGACACCAGGCAGAT	RT-qPCR
cpxP-R	TACCCGGCCAGCACCATA	RT-qPCR
gfp-F	TCAAGGACGACGGGAACT	RT-qPCR
gfp-R	ACTTGTGGCCGAGGATGT	RT-qPCR
16sRNA -F	GCCCAGGTAAGGTTCTTC	RT-qPCR
16sRNA -R	GGTGTAGCGGTGAAATGC	RT-qPCR
proA-F	CTGGACAGCGGGCTGAAGCT	RT-qPCR
proA -R	TCACTGCGTTGCCGGTTTT	RT-qPCR
proB-F	CGCGGTGGAAGCGATGAT	RT-qPCR
proB -R	CGCTGATTTCCTGCGAGTGG	RT-qPCR
proC-F	TGCTGGGTCGATGACGAAA	RT-qPCR

proC -R	CGCCTCCATAAACAGGAAGAAA		RT-qPCR
pip-F	GGAGACGGTCACGCTGTTG		RT-qPCR
pip-R	GCTTTCCAGGAAGCCCAGAT		RT-qPCR
serB-F	CGATGGGTTGGCATGTGG		RT-qPCR
serB-R	GCAGCGTATCGGCTTTGAACT		RT-qPCR
serC-F	TCGCCTGGTACTTGTCCG		RT-qPCR
serC-R	TTGGCCTGATTGCGTTTT		RT-qPCR
metH-F	CGATGTACGGTTCCAGGTAGG		RT-qPCR
metH-R	TTCGGCGAGGGCAAGATG		RT-qPCR
speD-F	CAACCGGCTGACCGAGAT		RT-qPCR
speD-R	GGGTGTTCCGACGTATCG		RT-qPCR
gpmA-F	TCCGCTTTGTTCAGACCC		RT-qPCR
gpmA-R	TTGACTTCGCTTACACCTCC		RT-qPCR
gapA-F	TTACAGCCCGCGTTGTTCC		RT-qPCR
gapA-R	AAAGTGCTCGCCTGGTATGAC		RT-qPCR
pykF-F	CATCGGACGCTTCGAGGAT		RT-qPCR
pykF-R	CGAGCACCTGAAAGCCCAC		RT-qPCR
aldB-F	GGCGAGGTGTTGGTGAAGTAG		RT-qPCR
aldB-R	GGTTTCGTTCCGTAAACGCTAT		RT-qPCR
P-*pig*P$_{961}$ F	CGCGGATCCGACGGAACCGATGCACAAC	*BamH* I	PCR
P-*pig*P$_{961}$ R	CCCAAGCTTGACGAACTCCGCCATTGG	*Hind* III	PCR
P-*pig*P$_{438}$ F	GAAGATCTCGAATCGTCTTGCTGGTTAAC	*BamH* I	PCR
P-*pig*P$_{438}$ R	GCTCTAGAGACGAACTCCGCCATTGG	*Hind* III	PCR
P-*pig*P$_{263}$ F	CGCGGATCCCAAAAAACAATAGCCAATCTAACC	*BamH* I	PCR
P-*pig*P$_{263}$ R	CCCAAGCTTGACGAACTCCGCCATTGG	*Hind* III	PCR
P-*pig*P$_{219}$ F	CGCGGATCCAAGATTTTTATTATATGTAAATTTT	*BamH* I	PCR
P-*pig*P$_{219}$ R	CCCAAGCTTGACGAACTCCGCCATTGG	*Hind* III	PCR
P-*pig*P$_{203}$ F	CGCGGATCCGTAAATTTTTGTGTATAAATAACAC	*BamH* I	PCR
P-*pig*P$_{203}$ R	CCCAAGCTTGACGAACTCCGCCATTGG	*Hind* III	PCR
P-*pig*P$_{71}$ F	CGCGGATCCTTGAGTTTTGGCCACCTCC	*BamH* I	PCR
pkk232-8 F	TTTTTTTAAGGCAGTTATTGGTGC		MP
pkk232-	TTTAGCTTCCTTAGCTCCTGAAAAT		MP

8 R			
GFP F	caggagctaaggaagctaaaATGAGTAAAGGAGAAGAACTTTTCACTG		MP
GFP R	caataactgccttaaaaaaaTTATTTGTATAGTTCATCCATGCCATG		MP
P*pig* F	cagcaaatgggtcgcggatccTTTTTCCTCCGGAATGCTCC	*BamH* I	VPA
P*pig* R	ttactcatGACGAACTCCGCCATTGGG		VPA
gfp(P) F	gcggagttcgtcATGAGTAAAGGAGAAGAACTTTTCACTG		VPA
gfp(P) R	gcaagcttgtcgacggagctcTTATTTGTATAGTTCATCCATGCCATG	*Sac* I	VPA

RS: Restriction sites, EOC: Expression, overexpression and complementation, FUHA: PigF up homologous arm, FDHA: PigF down homologous arm, RUHA: CpxR up homologous arm, RDHA: CpxR dowm homologous arm, VPA: Verify that the promoter is affected, MP: modification of plasmid

Supplementary Figures

Fig. S1

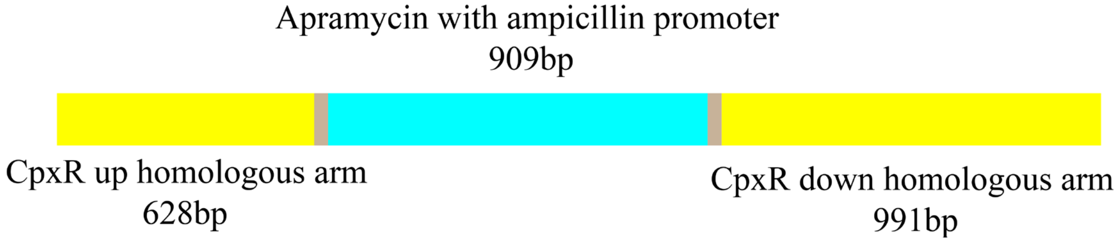

Supplementary Figure 1. The schematic of *cpxR* knockout. Left yellow bar represented *cpxR* up homologous arm (628bp) and right yellow bar represented *cpxR* down homologous arm (991bp). Middle blue bar epresented resistance marker gene apramycin resistant gene with ampicillin promoter. Left grey bar and right grey bar represented upstream sequence (9bp) and downstream sequence (9bp) of *cpxR* gene, respectively.

Fig. S2

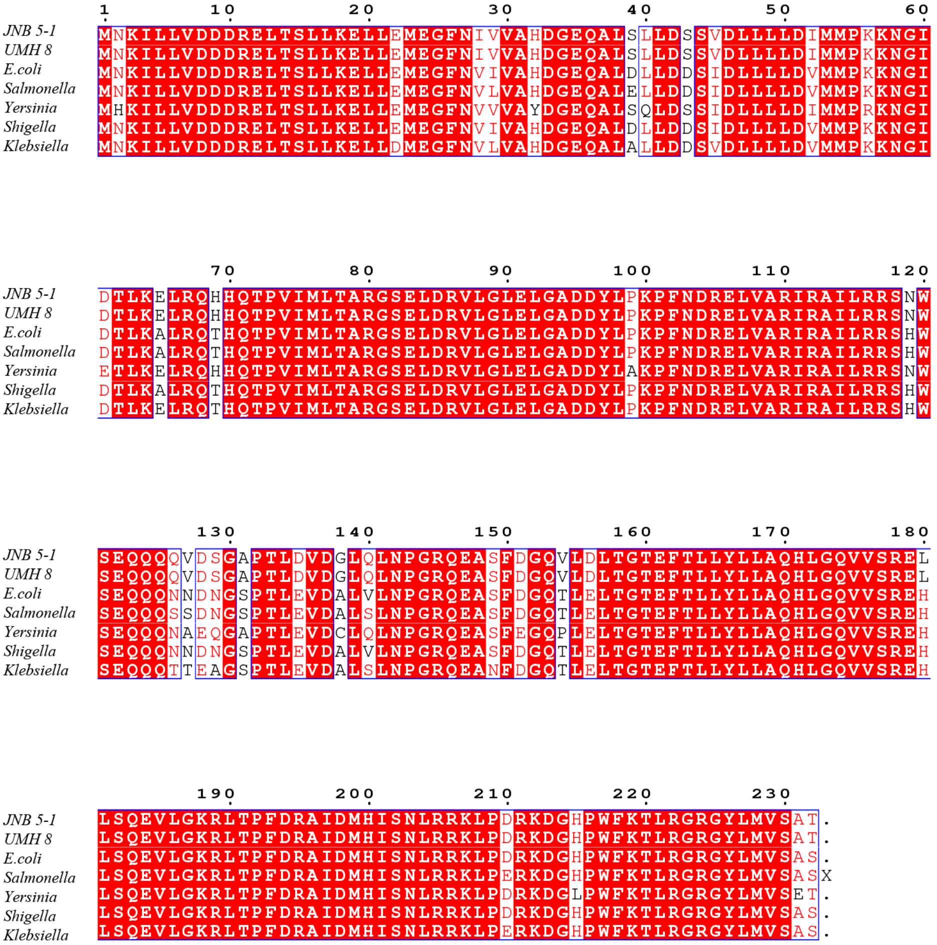

Supplementary Figure 2. Multiple sequence alignment of *cpxR* among the *Enterobacteriaceae*. Red region is high conved among the *Enterobacteriaceae.* Amino acid sequences in the figure are in the NCBI used for analysis of conservation in *CpxR* in *serratia marcescens*： CP018927.1 (*serratia marcescens* UMH8), NC_000913.3 (*Escherichia coli* K12), NC_003197.2 (*Salmonella enterica* LT2), NC_003143.1 (*Yersinia pestis CO92*), NC_007606.1 (*Shigella dysenteriae Sd197*), NZ_CP011636.1 (*Klebsiella oxytoca*).

Fig. S3

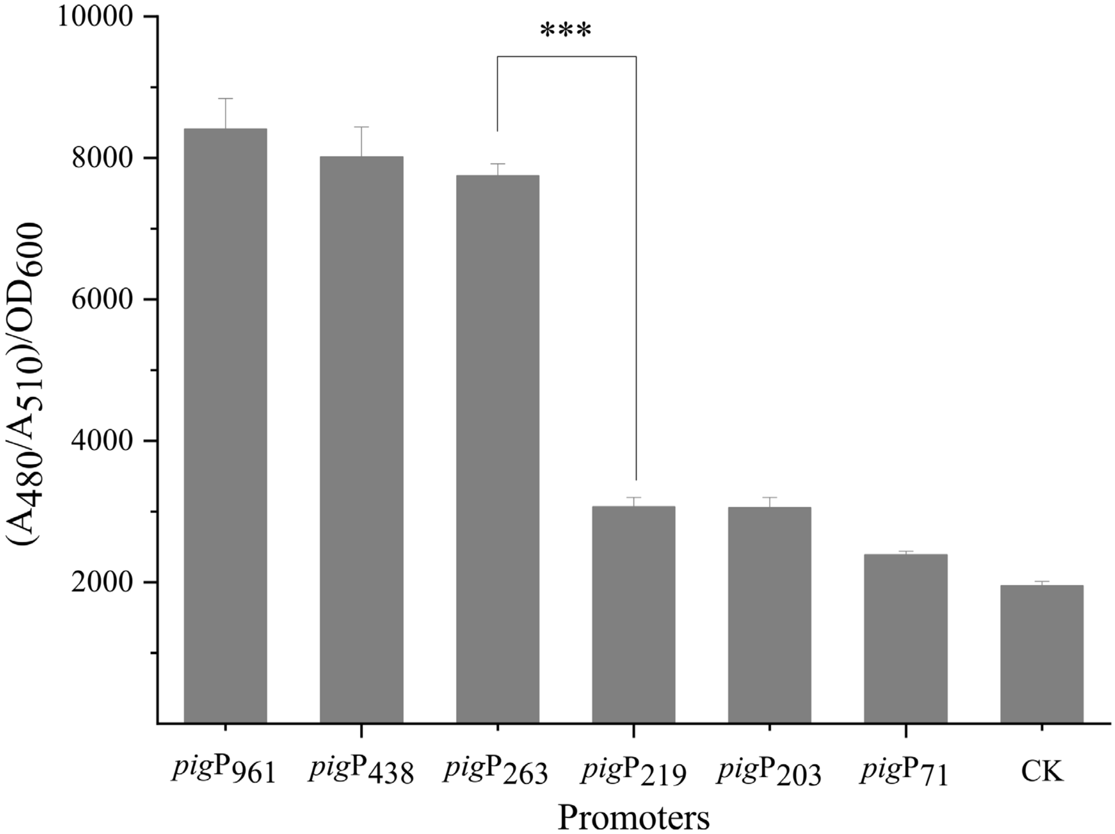

Supplementary Figure 3. Characterization of promoter activity at different lengths in GFP fluorescence intensity. Promoters at different lengths are fused to pKK 232-8-*gfp*. Means ± S.D. from three independent experiments are shown. Significant differences in activity by one-way ANOVA , ***(p<0.001).

Fig. S4

Serratia marcescens strain UMH8	TAAATAAACA**TTTATTTACATTTACC**ATTACTTTGC
Serratia sp. YD25	TAAATAAACA**TTTATTTACATTTACC**ATTACTTCGC
Serratia marcescens subsp ATCC 13880	TAAATAAACA**TTTATTTACATTTGCC**ATTACTGCGC
Serratia marcescens WW4	TAAATAAACA**TTTATTTACATTTGCC**ATTACTGCGC
Serratia marcescens strain B3R3	TAAATAAACA**TTTGTTTACATTTGCC**ATTACTGCGC
Serratia marcescens strain U36365	TAAATAAACA**TTTGTTTACATTTGCC**ATTACTGCGC
Serratia marcescens strain EL1	CAAATAAACA**TTTATTTACATTTGCC**ATTACTGCGC
Serratia marcescens strain KS10	CAAATAAACA**TTTATTTACATTTGCC**ATTACTGCGC
Serratia marcescens strain S2I7	CAAATAAACA**TTTATTTACATTTGCC**ATTACTGCGC
Serratia sp. FS14	CAAATAAACA**TTTATTTACATTTGCC**ATTACTGCGC
Serratia marcescens AS-1	CAAATAAACA**TTTATTTACATTTGCC**ATTACTTTAC
Serratia marcescens strain SGAir0764	CAAATAAACA**TTTATTTACATTTACC**ATTACTTTGC
Serratia marcescens strain N4-5	CAAATAAACA**TTTATTTACATTTACC**ATTACTTTGC

Supplementary Figure 4. CpxR binding site motif is highly conserved in *pig* gene cluster promoter. Red and bold sequence is binding region that predicte CpxR binding site motif interacted. DNA sequences in the figure are in the NCBI used for analysis of interaction between CpxR binding site motif and pig gene cluster promoter in *serratia marcescens*： CP018927.1 (*serratia marcescens* UMH8), CP016948.1 (*serratia sp.* YD25) , CP041233.1 (*serratia marcescens subsp.* ATCC 13880), CP003959.1 (*serratia marcescens* WW4), CP013046.2 (*serratia marcescens* strain B3R3), CP016032.1 (*serratia marcescens* strain U36365), CP027796.1 (*serratia marcescens* strain EL1), CP027798.1 (*serratia marcescens* strain KS10), CP021984.1 (*serratia marcescens* strain S217), CP005927.1 (*serratia sp.* FS14), AP019009.1 (*serratia marcescens* AS-1), CP027300.1 (*serratia marcescens* strain SGAir0764), CP031316.1 (*serratia marcescens* strain N4-5).

References:

[1]. de Lorenzo, V., et al., Mini-Tn5 transposon derivatives for insertion mutagenesis, promoter probing, and chromosomal insertion of cloned DNA in gram-negative eubacteria. J Bacteriol, 1990. 172(11): p. 6568-72.

[2]. You, J., et al., Regulatory protein SrpA controls phage infection and core cellular processes in Pseudomonas aeruginosa. Nature Communications, 2018. 9(1).
